# A novel enemy of cancer: recent investigations into protozoan anti-tumor properties

**DOI:** 10.3389/fcimb.2023.1325144

**Published:** 2024-01-11

**Authors:** Zhi Zheng, Xiao Lu, Dong Zhou, Xu-feng Deng, Quan-xing Liu, Xiao-bing Liu, Jiao Zhang, Yan-qi Li, Hong Zheng, Ji-gang Dai

**Affiliations:** Department of Thoracic Surgery, Xinqiao Hospital, Army (Third Military) Medical University, Chongqing, China

**Keywords:** protozoa, protozoan components, anti-tumor, immune response, angiogenesis

## Abstract

Cancer remains a significant global health issue, despite advances in screening and treatment. While existing tumor treatment protocols such as surgery, chemotherapy, radiotherapy, targeted therapy, and immunotherapy have proven effective in enhancing the prognosis for some patients, these treatments do not benefit all patients. Consequently, certain types of cancer continue to exhibit a relatively low 5-year survival rate. Therefore, the pursuit of novel tumor intervention strategies may help improve the current effectiveness of tumor treatment. Over the past few decades, numerous species of protozoa and their components have exhibited anti-tumor potential via immune and non-immune mechanisms. This discovery introduces a new research direction for the development of new and effective cancer treatments. Through *in vitro* experiments and studies involving tumor-bearing mice, the anti-tumor ability of *Toxoplasma gondii*, *Plasmodium*, *Trypanosoma cruzi*, and other protozoa have unveiled diverse mechanisms by which protozoa combat cancer, demonstrating encouraging prospects for their application. In this review, we summarize the anti-tumor ability and anti-tumor mechanisms of various protozoa and explore the potential for their clinical development and application.

## 1 Introduction

Cancer comprises a group of diseases that can rapidly and uncontrollably generate abnormal cells, afflicting any tissue or organ of the human body. Despite decades of research and progress in cancer screening and treatment, the overall morbidity and mortality rates remain high, with approximately 20 million new cancers and nearly 10 million deaths each year (Sung et al., 2021). Surgery, radiotherapy, and chemotherapy are the most mature and widely used methods of cancer treatment. Although these traditional treatment methods currently occupy a dominant position in cancer treatment, their application is limited by factors such as tumor grade, stage, and patient tolerance. Moreover, their application comes with significant side effects. Surgical treatments can directly harm bodily structures, radiation therapy may damage normal cells or induce secondary cancers, and chemotherapy drugs can adversely affect healthy organs (Chon et al., 2012; Fillon, 2019). Therefore, there is a growing need to explore innovative cancer treatments beyond these conventional methods.

Immunotherapy, which aims to strengthen the weakened immune system to better combat cancer, has emerged as a promising alternative to traditional treatment methods (Tan et al., 2020). This field has witnessed rapid development, with notable achievements translating to clinical cancer treatment. Immune checkpoint inhibitors, for instance, work by lifting the constraints on the immune response to enhance tumor immunity (Ribas and Wolchok, 2018). Chimeric antigen receptor T cell immunotherapy employs genetic engineering to equip T cells with the ability to target cancer cells (June et al., 2018). However, these immunotherapies can lead to autoimmune reactions that harm normal tissues, and some patients exhibit poor responsiveness. Meanwhile, drug therapy for cancer, including targeted drugs that focus on genes or proteins related to cancer progression, has also made great progress (Okuyama et al., 2023). Some mature drugs from other fields, such as anti-parasitic drugs (Li Y. Q. et al., 2021), have been explored for their anti-tumor potential. Nevertheless, challenges like drug resistance and high costs impede the progress of these therapies. Both traditional and emerging cancer treatments have obvious limitations and are not poised for immediate breakthroughs. This underscores the pressing need to explore and develop new methods for cancer treatment.

Parasites have long been associated with pathogenic and harmful effects. However, recent studies have unveiled potential non-negative associations between parasitic infections and various nonparasitic diseases. For example, *Trichuris suis* infection can alleviate active ulcerative colitis in clinical patients (Summers et al., 2005), while *Toxoplasma gondii* (*T. gondii*) infection can reduce the size of cerebellar infarction in the mouse model (Arsenijevic et al., 2007). These findings suggest that strategic use of parasites might offer therapeutic benefits in the context of multiple diseases, with cancer being one of the most extensively researched areas (Guan et al., 2019). According to epidemiological data, the infection rate of certain parasites is found to be inversely proportional to tumor mortality rates (Qin et al., 2017). This interesting finding suggests that individuals infected with particular parasites may have a reduced likelihood of dying from cancer. It is possible that a competitive relationship between parasites and tumors exists within the host. This competition hints at the potential of parasitic infections as a means to combat cancer. When parasites infect the human body, they trigger a robust immune response (Dupont et al., 2012). Simultaneously, the immune response against tumor cells is either activated or reactivated in individuals with cancer, which constitutes one of the primary mechanisms through which parasites exert their anti-cancer effects (Darani and Yousefi, 2012). Furthermore, specific parasite components, including lysates and vital functional proteins, can hinder cancer progression through non-immune mechanisms, such as promoting the release of anti-tumor cytokines (Wang et al., 2019) and resisting tumor angiogenesis (Ramírez-Toloza et al., 2016) *in vivo*. Undoubtedly, other microorganisms such as viruses and bacteria have shown promise in anti-tumor therapy as immune boosters or vectors (Li et al., 2022). However, protozoa possess distinct advantages in this context. Firstly, as eukaryotes, protozoa have the capability to more accurately express human proteins, a feat that is beyond the reach of prokaryotes or non-cellular organisms. Building upon this, protozoa can express tumor antigens and subsequently stimulate targeted anti-tumor immunity within the host, thereby enhancing the tumor-specific therapeutic effect—an essential requirement for effective anti-tumor therapy.

Protozoa, being part of parasites, do pose potential harm to the human body, and their application in treating human tumors faces considerable ethical and regulatory challenges. Encouragingly, significant progress has been made in the research on attenuating various strains of protozoa (Fox and Bzik, 2010). Over a decade ago, researchers successfully conducted clinical studies involving the injection of nonreplicating sporozoites into human subjects (Seder et al., 2013), which provided evidence that attenuated strains can be tolerated by the human body. These advancements pave the way for further exploration of protozoa as a promising novel means of combating tumors. Overall, protozoa present themselves as a viable avenue for future investigation into novel anti-tumor therapies, despite the inherent challenges associated with their application in treating human tumors.

In this review, we focus on *T. gondii*, *Plasmodium*, *Trypanosoma cruzi* (*T. cruzi*), *Leishmania*, and *Eimeria*, among the well-studied protozoans, to explore their anti-tumor capabilities and mechanisms, as well as their constituent components. This examination aims to consolidate current research progress, identify existing gaps, and guide future research and clinical applications in the realm of protozoan-based anti-tumor therapies.

## 2 T. gondii

**Figure 1:**
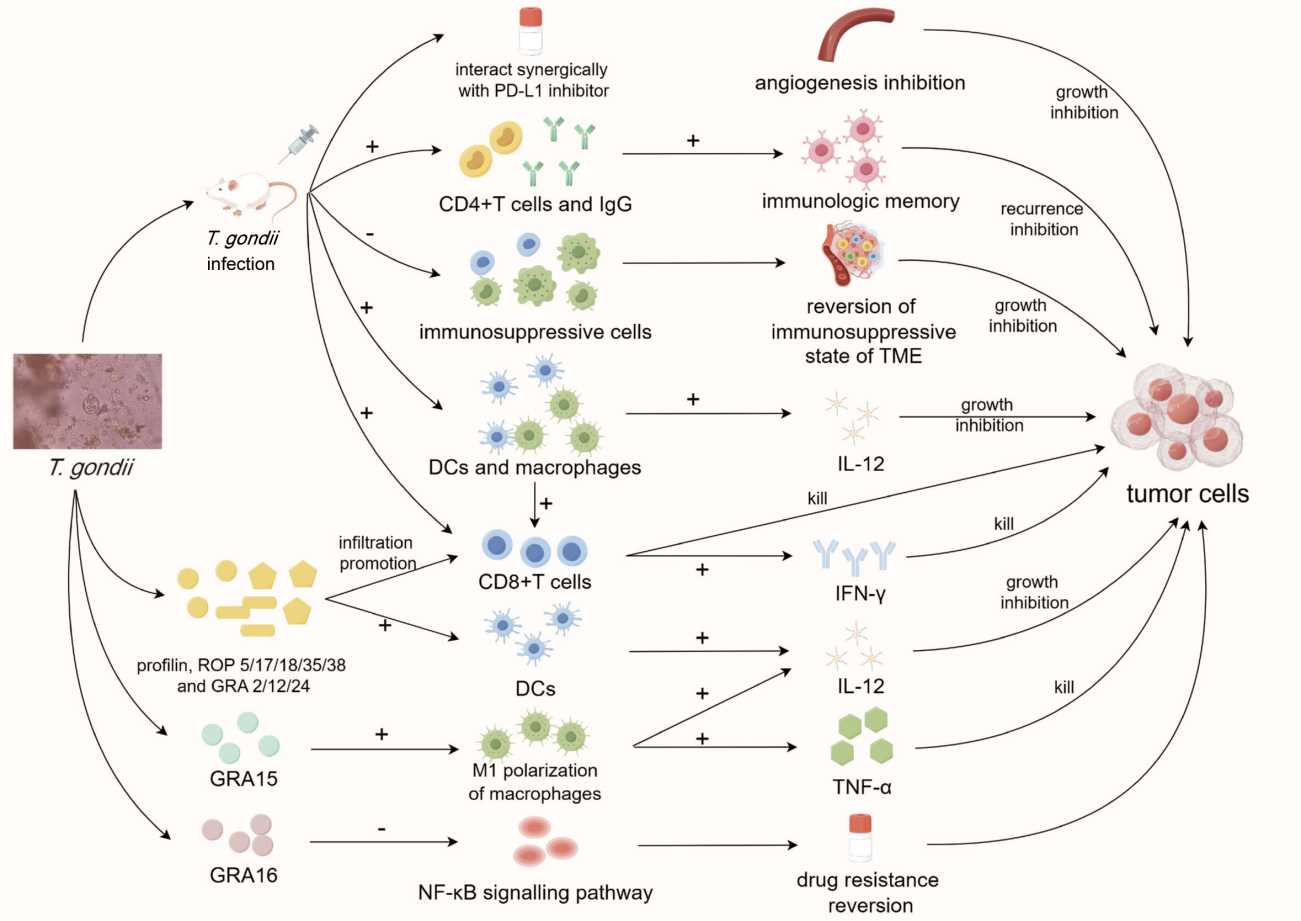
Diagram of anti-tumor mechanisms of *T. gondii*. Infection of *T. gondii* can reverse the immunosuppressive state of tumor microenvironment and inhibit tumor angiogenesis, and its components profilin, ROP and GRA can also inhibit tumor progression through multiple immune and non-immune pathways. *T. gondii*, *Toxoplasma gondii*; PD-L1, programmed death ligand-1; CD4/8, cluster of differentiation 4/8; IgG, immunoglobulin G; TME, tumor microenvironment; DCs, dendritic cells; IL-12, interleukin-12; IFN-γ, interferon-γ; TNF-α, tumor necrosis factor-α; ROP, rhoptry; GRA, granule; NF-κB, nuclear factor kappa-B.

*T. gondii* is an obligate intracellular protozoan parasite that infects nearly a third of the world’s population. This parasite is typically transmitted through the consumption of undercooked meat, ingestion of water contaminated with oocysts, or contact with pet (particularly cats) feces (Montoya and Liesenfeld, 2004). *T. gondii* infections often remain latent, with infected people usually exhibiting minimal to no symptoms. However, when the immune system weakens or is compromised, *T. gondii* can lead to severe symptoms affecting the brain, eyes and muscles (Dupont et al., 2012). Remarkably, based on the fact that *T. gondii* infection can stimulate the immune system to produce a series of powerful immune responses (Dupont et al., 2012), studies have found that the tumor progression can be greatly slowed down and the prognosis is obviously improved after infection of *T. gondii* in tumor-bearing mouse models (Seyedeh et al., 2015; Wang et al., 2019). Additionally, several studies have highlighted the close relationship between *T. gondii* infection and cancer. For example, *T. gondii* exhibits glucose-independent growth in its tachyzoite stage, which shares similarities with the metabolic characteristics of tumor growth (Nitzsche et al., 2017). Moreover, anti- *T. gondii* antibodies in tumor-bearing mice have been shown to specifically attach to tumor cells (Mohamadi et al., 2019). These findings suggest that *Toxoplasma* has great potential to induce the organism to fight against tumors (**Figure 1**). Certainly, the precise relationship between *T. gondii* and cancer remains uncertain (El Skhawy and Eissa, 2023). *T. gondii*, a common parasite capable of infecting the human body and causing harm, has been the subject of conflicting research findings regarding its association with tumor occurrence and development, particularly in brain tumors (Jung et al., 2022). Some researchers propose that *T. gondii* primarily promotes tumor progression within the human body, while others argue that *T. gondii*, particularly during asymptomatic infections, can impede tumor growth. Both sides present compelling evidence to support their respective viewpoints. In this context, our focus lies in elucidating the current research findings pertaining to the potential anti-tumor effects of *T. gondii*.

### 2.1 Anti-tumor effects of wildtype or genetically modified *T. gondii* infection

*T. gondii*, known for its ability to trigger a potent Th1 immune response, has garnered considerable interest regarding its potential as an anti-tumor agent in the past century. In the early 1980s, Conley et al. found that mice infected intraperitoneally with *T. gondii* experienced a significant increase in necrotic areas within central nervous system tumors, effectively impeding tumor progression (Conley and Remington, 1977; Conley, 1980). Subsequently, researchers successfully infected mice bearing Lewis lung cancer with orally administered *T. gondii* strains, resulting in robust immune responses that had a significant impact on tumor growth (Kim et al., 2007). Further investigations explored the direct impact of *T. gondii* on tumor cells by co-culturing tachyzoites with Her2/Neu-expressing mammary cancer cells or hepatoma carcinoma H7402 cells (Wang and Gao, 2016; Atalay Şahar et al., 2020). In the co-culture process, *T. gondii* could invade Her2/Neu-expressing mammary cancer cells and replicate in large numbers, leading to the destruction of tumor cells, or down-regulate the expression of cyclinB1 and cdc2 in hepatoma carcinoma H7402 cells to promote apoptosis. These early explorations initially confirmed the anti-tumor potential of *T. gondii*. However, additional studies are needed to refine the toxicity and reveal the anti-tumor mechanisms of *T. gondii*.

The primary challenge in applying *T. gondii* for human cancer treatment lies in the virulence of the strains. As mentioned above, *T. gondii* infection is more harmful to immunocompromised patients, and cancer often weakens the immune systems of patients (Xia et al., 2021), especially in advanced cases. However, the invasive activity of live *T. gondii* is important for triggering the anti-tumor response. Therefore, the strain must be attenuated while preserving its invasiveness. Researchers have made substantial efforts to understand the source of *T. gondii* virulence and reduce or eliminate it. For example, Barbara et al. proved that *T. gondii* needs *de novo* synthesis of pyrimidine for its virulence (Fox and Bzik, 2002). Knocking out the orotidine-5’-monophosphate decarboxylase gene of *T. gondii* rendered the strain unable to replicate normally without uracil supplementation, leading to the creation of an avirulent non-replicating vaccine strain (CPS) (Fox and Bzik, 2010). Other methods, such as knocking out other key genes and applying gamma radiation, have also been found to be effective in reducing the virulence of *T. gondii* without affecting its immunogenicity after infection (Hiramoto et al., 2002; Xia et al., 2018). By working with attenuated or non-toxic *T. gondii* strains, researchers can explore anti-tumor effects without the interference of toxicity, resulting in more accurate results that closely resemble actual treatment conditions.

The human immune system has an inherent mechanism for eliminating tumor cells. However, tumor-induced immunosuppression often severely weakens this clearance process, causing immune cells to fail to target tumors and resulting in immune evasion (Bayne et al., 2012). The tumor microenvironment (TME) contains myeloid cells such as dendritic cells (DCs) and macrophages, which play a pivotal role in immunosuppression and tumor progression. Attenuated *T. gondii* tends to preferentially invade and activate these cells, regulating their functions to reverse the immunosuppressive state within the TME (Fox et al., 2013; Chen et al., 2022). In recent years, CPS, as an attenuated strain with relatively mature technology, has been applied in several anti-tumor experiments. In mouse models of pancreatic and ovarian tumors, intraperitoneal CPS treatment successfully reduced the proportion of immunosuppressive cells (tumor-associated macrophages and Treg cells) within the TME (Sanders et al., 2015). Simultaneously, it stimulated DCs and macrophages to express more CD80/86 co-stimulatory molecules and produce more interleukin-12 (IL-12) (Baird et al., 2013a), thus countering the immunosuppressive environment around tumor cells and triggering a range of anti-tumor immune responses. This led to the increased infiltration of diverse immune cells, including CD8^+^ T cells, which produced more IFN-γ and facilitated specific tumor cell clearance (Baird et al., 2013b; Sanders et al., 2015). Intratumoral treatment of CPS can increase the secretion of IL-12 and IFN-γ in TME and inhibit the tumor growth of breast cancer in a mouse model (Xu et al., 2021). In addition, Bahwal et al. found that the antitumor efficacy of anti-PD1 antibody in pancreatic cancer bearing mice could be significantly enhanced by intratumoral injection of CPS (Bahwal et al., 2022). In studies involving melanoma and pancreatic tumors, intraperitoneal CPS treatment activated CD4^+^ T cells and elevated tumor-specific IgG antibody titers, which, while exerting less pronounced inhibitory effects on primary tumors (Sanders et al., 2016), played essential roles in tumor prognosis, immune memory, and long-term protective effects against tumor recurrence (Baird et al., 2013b). Other attenuated *T. gondii* strains, such as lactate dehydrogenase-deficient strains (LDH1/2 knockout), activate a significantly strong Th1 immune response in mouse models after intratumoral injection (Li Y. et al., 2021). Moreover, intratumoral injection of ΔGRA17 strains were found to interact synergically with programmed cell death 1 ligand 1 checkpoint inhibitors to improve anti-tumor activity in several kinds of tumors (Zhu et al., 2021). Additionally, oral feeding of the gamma radiation-attenuated strains could promote the secretion of IL-12 and IFN-γ in tumor-bearing mice (Hafez et al., 2020a; Hafez et al., 2020b). All these effects contribute to the elimination of tumor cells and inhibition of progression.

Furthermore, Hunter et al. demonstrated that *T. gondii* systematically inhibits angiogenesis *in vivo* during acute toxoplasmosis, which is a classic non-immune target in tumor therapy (Hunter et al., 2001). *T. gondii* can also be engineered as a live vaccine vector (Zou et al., 2011), stimulating the immune system to produce a long-lasting anti-tumor immune response by carrying tumor-related antigens with the characteristics of long-term latent infection. Such treatment would not only achieve the goal of eliminating tumor cells but also prevent tumor recurrence in long terms in condition of vaccine efficacy. The miRNA levels of mice fed *T. gondii* cysts have also been confirmed to undergo significant changes, represented by miR-429-3p, miR-145a-5p, miR-211-5p, miR-31-3p, and miR-135a-5p, which target tumor related genes TNF receptor superfamily member 11b, large tumor suppressor kinase (Lats) 2 and Lats (Wang et al., 2022). In terms of bioinformatics, transcriptome analysis of mice infected with *T. gondii* revealed significant changes in the expression of several inflammation-related genes (such as CD74, IFNG and CXCL9) (Caner, 2021) and key signaling pathways (such as MAPK signaling pathway, RAP1 signaling pathway and RAS signaling pathway) (Lu et al., 2019). These changes potentially exert an inhibitory influence on the tumor cells existing in the host, thereby revealing the anti-tumor potential of *T. gondii* from another perspective. In terms of prognosis, titer of anti-*T. gondii* IgG was found to be associated with the 10-year survival rate of breast cancer patients, and patients with high titers of anti-*T. gondii* IgG, IL-17 and IL-9 had a HR of 0.29 (Ye et al., 2023).

### 2.2 Anti-tumor potential of *T. gondii* components

In the early stages of research, Suzuki et al. injected formalin-fixed *T. gondii* organisms into the tumor of Lewis tumor bearing mice, resulting in significant inhibition of tumor growth (Suzuki and Kobayashi, 1985). Additionally, it was noted that using *T. gondii* lysate antigen alone could reverse multidrug resistance in cancer cells *in vitro* (Varga et al., 1999) or inhibit tumor growth when injected intratumorally in tumor-bearing mice (Darani et al., 2009; Pyo et al., 2014). This indicates that the anti-tumor ability of *T. gondii* may not only be solely attributed to its cellular invasion but may also involve the regulatory impact of its constituent components on the host organism. However, the specific *T. gondii* components responsible for the anti-tumor response remained unidentified. Therefore, further studies are needed to identify the specific effective proteins or cytokines within *T. gondii* that drive its anti-tumor response.

Soluble tachyzoite antigen (STAg) was shown to effectively stimulate DC maturation *in vitro*, promote its secretion of IL-12p70, and enhance its ability to activate lymphocytes. When these mature DCs are injected into the tumor periphery of tumor bearing mice, tumor growth is significantly inhibited (Motamedi et al., 2009; Boghozian et al., 2015). Profilin, an immunodominant protein in STAg, can greatly promote the infiltration of CD4^+^ and CD8^+^ T cells into the TME after intraperitoneal injection into tumor-bearing mice (Payne et al., 2021). The exosomes produced by DCs infected with *T. gondii* can promote M1 macrophage polarization in the bloodstream by transporting miR-155-5p, and inhibit the suppressor of cytokine signaling 1 (SOCS1) and signal transduction and transcription activating factor (STAT3) signaling pathways to regulate MDSC levels, thereby inhibiting tumor progression in colorectal cancer bearing mice (Lu et al., 2022; Zhu et al., 2022). Two important organelles of *T. gondii.*, apical rhoptry (ROP) (Dlugonska, 2008) and dense granule (GRA) (Panas and Boothroyd, 2021), play essential roles in the invasion of host cells and normal replication. Barbara et al. revealed that ROP5, ROP17, ROP18, ROP35, ROP38, GRA2, GRA12, and GRA24 play key roles in the activation of CD8α^+^ DCs, CD4^+^, and CD8^+^ T cells, and the stimulation of the IL-12/IFN-γ Th1 axis (Fox et al., 2016). GRA15, when secreted by *T. gondii* into host cell cytoplasm, effectively activates stimulator of interferon protein genes and stimulates innate immunity (Wang et al., 2019). Additionally, transfecting macrophage to express GRA15 leads to M1 polarization and promotes the secretion of TNF-α and IL-12 in hepatocellular carcinoma tumor-bearing mice (Li et al., 2017). GRA16 has been found to up-regulate the expression of protein phosphatase 2A regulatory subunit (B55PP2A-B55) and inhibit the phosphorylation of AKT, leading to NF-κB inactivation and the reversal of drug resistance in cancer cells (Seo et al., 2020). Subsequent research by the same group has shown that after transgenic expression of GRA16 in colorectal cancer cell line HCT116, it can promote the activation of tumor suppressor phosphatase and tensin homolog (PTEN), leading to telomerase inactivation and achieving the effect of promoting tumor cell apoptosis *in vitro* (Seo et al., 2022). Additionally, GRA8 can promote the efficacy of tumor-targeted drugs by interacting with mitochondrial protein ATP synthase F1 subunit alpha/sirtuin-3 both *in vitro* and *in vivo* (Kim et al., 2020). In conclusion, several components within *T. gondii* can exert anti-tumor effects through a variety of immune and non-immune pathways. According to the recent research progress, it is worth mentioning that the components of *T. gondii* have good potential for anti-tumor applications. Eissa et al. demonstrated that mice immunized with autoclaved *Toxoplasma* vaccine (ATV) produced 13.3% inhibition of Ehrlich solid carcinoma (ESC) genesis and confirmed the existence of at least four pairs of common antigens between ATV and ESC (Eissa et al., 2023). Subsequent results showed that ATV and cyclophosphamide synergistically exerted a stronger tumor immune response and anti-angiogenesis effect in ESC-bearing mice, indicating that ATV has the potential to be a tumor vaccine or cancer drug adjuvant (Ismail et al., 2023).

Among protozoa exhibiting potential anti-tumor properties, *T. gondii* has been extensively studied and holds significant promise for application. However, current research efforts have not extensively explored its tumor-targeting capabilities, limiting its potential use in treating non-solid tumors or multiple solid tumors. Furthermore, while the ROP and GRA proteins have received considerable attention, the anti-tumor potential of other important components of *T. gondii* remains largely unexplored. It is important to note that existing anti-tumor research on *T. gondii* is largely constrained by previous technologies. However, recent advancements in single-cell technology, machine learning, and multi-omics approaches may provide new impetus for furthering the study of *T. gondii* in anti-tumor research.

## 3 Plasmodium

**Figure 2:**
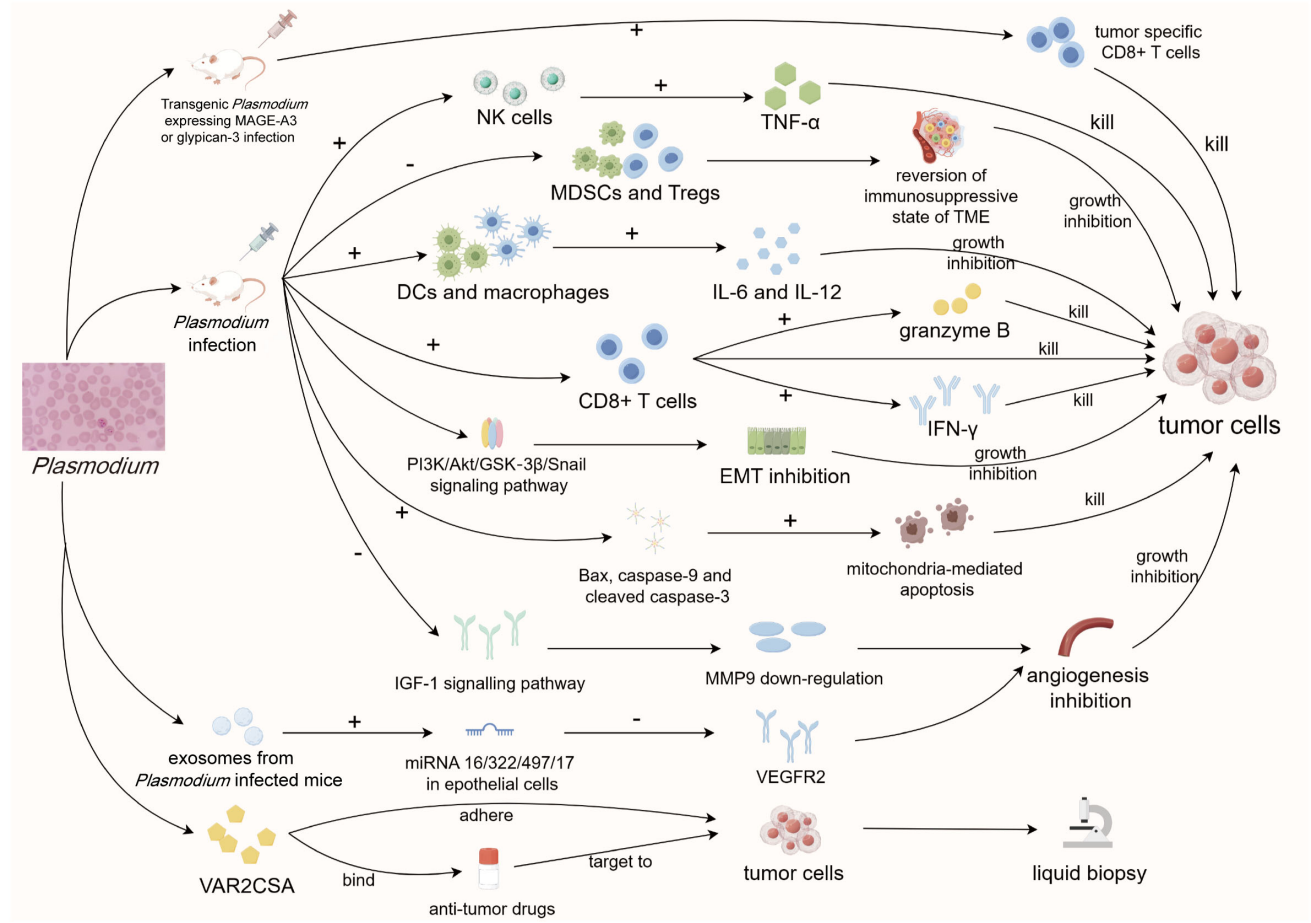
Diagram of anti-tumor mechanisms of *Plasmodium*. *Plasmodium* infection can stimulate the immune system through various pathways to reverse tumor immune suppression and activate multiple signaling pathways to indirectly inhibit tumor progression. Meanwhile, its important component VAR2CSA can specifically target and adhere to tumor cells, which can be used for tumor liquid biopsy and the development of targeted drugs. MAGE-A3, Melanoma-associated antigen 3; CD8, cluster of differentiation 8; NK cells, natural killer cells; TNF-α, tumor necrosis factor-α; MDSCs, myeloid-derived suppressor cells; Tregs, regulatory T cells; TME, tumor microenvironment; DCs, dendritic cells; IL-6/12, interleukin-6/12; IFN-γ, interferon-γ; EMT, epithelial-mesenchymal transition; IGF-1, insulin-like growth factor 1; MMP9, matrix metalloprotease 9; VEGFR2, vascular endothelial growth factor receptor 2; CSA, chondroitin sulfate A.

Malaria is a disease primarily transmitted by *Anopheles* mosquitoes that causes severe fever and anemia and is mainly endemic in Africa (approximately 85% of the cases) and Southeast Asia. Of the more than 120 *Plasmodium* strains identified, only six can infect humans (Ashley et al., 2018). *Plasmodium falciparum* and *Plasmodium vivax* are the primary agents responsible for malaria transmission, and extensive research has been conducted on these strains. Interestingly, over several decades of malaria research, comparative data analysis has revealed that the effects of *Plasmodium* infection are not uniformly negative. For example, by using generalized additive mixed model to calculate the epidemiologic data from 56 countries, Qin et al. demonstrated that the malaria prevalence is inversely proportional to the mortality of tumors (Qin et al., 2017; Wyss et al., 2020), and several antimalarial drugs have also demonstrated anti-tumor effects (Neznanov et al., 2009; Jung et al., 2018; Das and Kundu, 2021). These findings suggest a potential competitive relationship between *Plasmodium*, other parasites, and neoplasms within the host, as well as potential overlapping survival strategies, nutrient dependencies, and signaling pathways. Together, these findings imply that *Plasmodium* has the potential to stimulate the anti-tumor response of the host (**Figure 2**).

### 3.1 Anti-tumor effects of wildtype or genetically modified *Plasmodium* infection

In the mid-20th century, Trager et al. discovered that chickens displayed age-related resistance patterns to both *Plasmodium* and tumor type 1 of Rous (a chicken tumor), leading to subsequent experiments that confirmed an interaction between the two and revealed that *Plasmodium* significantly inhibited tumor growth (Trager and Mcghee, 1953). This finding paved the way for the exploration of *Plasmodium*’s potential in anti-tumor research. Unfortunately, due to limited resources at the time, no further investigations were conducted to elucidate the mechanism behind anti-tumor effects of *Plasmodium*.

When *Plasmodium* infects a host, it often triggers a robust combination of innate and acquired immune responses (Ing et al., 2006; Roetynck et al., 2006). Since *Plasmodium* may share some similarities with tumors, it is likely that the activated immune system contributes to the elimination of both tumors and parasites. For example, studies conducted by Chen et al. on a Lewis lung cancer (LLC) mouse model infected with *Plasmodium* revealed effective activation of both the innate and adaptive immune system. This included increased secretion of IFN-γ and TNF-α, greater numbers of activated NK cells, and higher levels of tumor-specific effector CD8^+^ T cells (Chen et al., 2011). These immune cell changes and alterations in immune-active substances are recognized as crucial contributors to tumor immunity. Our previous study proved that intravenous injection of attenuated *Plasmodium* can inhibit LLC growth and prolong the survival of tumor-bearing mice by promoting the secretion of IL-6 and IL-12 and inhibiting tumor angiogenesis (Deng et al., 2016). Similarly, in a triple-negative breast cancer mouse model, intraperitoneal infection of *Plasmodium* successfully promoted the infiltration of CD8^+^ T cells into the TME and the secretion of granzyme B. This process was accompanied by up-regulation of both co-stimulatory (CD40L, GITR, and OX-40) and co-inhibitory (PD-1, CTLA-4, TIM-3, and LAG3) immune checkpoints (Pan et al., 2021), further confirming the ability of *Plasmodium* treatment to effectively combat various types of tumors. In the TME, some immunosuppressive cells pose significant challenges to tumor immune clearance (Gattinoni et al., 2006; Serafini et al., 2006). These cells, including myeloid-derived suppressor cells (MDSCs) and regulatory T cells (Tregs), are closely related to the immune escape of tumor cells (Kusmartsev et al., 2000; Curiel, 2007). Eliminating the obstruction of these immunosuppressive cells is one of the main challenges facing tumor immunotherapy at present. Adah et al. demonstrated that *Plasmodium* infection in Lewis tumor-bearing mice significantly reduced the expression of enrichment factors such as GMCSF, GCSF, MCSF, IL-1β, IL-6, and IL-14 in MDSCs and Treg cells. This reduction effectively diminished the proportion of these two immunosuppressive cell types and reversed the immunosuppressive environment within the TME (Adah et al., 2019). Simultaneous intraperitoneal injection of *Plasmodium* and gemcitabine treatment in Lewis lung cancer mice showed a synergistic effect, significantly downregulating Snail protein and upregulating E-cadherin expression levels, thereby inhibiting EMT of tumor cells and greatly prolonging the survival of tumor bearing mice (Chen et al., 2023). However, solely relying on *Plasmodium* infection to broadly stimulate strong immune responses lacks specificity in combating cancer. This challenge is prevalent in many current studies focusing on parasitic anti-tumor approaches. To enhance anti-tumor precision and efficiency, it is crucial to target the immune response specifically against tumor cells.

In recent years, tumor vaccine research has garnered significant attention. While it has shown promising results, it also faces substantial challenges. The clinical application of anti-tumor vaccine must ensure both strong immunogenicity and long-term immune efficacy (Morse et al., 2021). Equally important is the safety of using these vaccines in the human body. Attenuated protozoa have emerged as a suitable vector for long-term chronic infection-based vaccines, meeting these requirements. However, the key challenge in many parasitic anti-tumor studies is the lack of specificity in fighting cancer, which is primarily due to the broad immune responses generated by *Plasmodium* infection. To address this, there is a need to focus on immune responses against tumor cells specifically, making anti-tumor interventions more precise and efficient. Our efforts, along with others in the field, have centered on creating transgenic *Plasmodium* strains that express recognized tumor-specific antigens, such as Melanoma-associated antigen 3 in non-small cell lung cancer cells (Dong et al., 2019) and glypican-3 in liver cancer cells (Liu et al., 2017). After infecting the corresponding tumor-bearing mice intravenously, the immune system successfully recognized tumor-specific antigens while mounting immune responses against *Plasmodium*. This process significantly promoted specific CD8^+^ T cell responses against tumor cells, thus inhibiting cancer progression.

In addition to activating the immune system, *Plasmodium* infection also combats tumors via non-immune mechanisms. For example, Liang et al. found that intraperitoneal injection of *Plasmodium* in hepatoma tumor-bearing mice could affect the PI3K/Akt/GSK−3β/Snail signaling pathway by down-regulating the expression of CC−chemokine receptor 10. This inhibition, in turn, hinders the epithelial-mesenchymal transition process of tumor cells and resists the recurrence and metastasis of liver cancer cells (Liang et al., 2021). Tumor-associated macrophages can produce matrix metalloprotease 9 (MMP9), a kind of enzyme responsible for tumor angiogenesis. Intraperitoneally injected *Plasmodium* has been shown to reduce MMP9 production by inhibiting IGF-1 signaling through the accumulation of hemozoin, thus inhibiting tumor angiogenesis *in vivo* (Wang et al., 2020). In addition, tumor cells from *Plasmodium*-infected mice exhibited increased expression of Bax, caspase-9, and cleaved caspase-3 proteins, while Bcl-2 expression was decreased, resulting in the promotion of mitochondria-mediated apoptosis (Yao et al., 2022). Unfortunately, the specific pathways regulating these non-immune mechanisms following *Plasmodium* infection remain unclear, necessitating further molecular studies to elucidate the mechanisms involved.

### 3.2 Anti-tumor properties of *Plasmodium* components

*Plasmodium* replicates within infected erythrocytes, causing the red blood cells to swell and become more susceptible to immune elimination. To avoid being detected by the immune system, the parasite expresses proteins on the erythrocyte membrane during replication, allowing host cells to adhere to the vasculature (Baruch et al., 1995). One such proteins, VAR2CSA, specifically adheres to chondroitin sulfate (CS) A on placental syncytial trophoblast cells (Ayres Pereira et al., 2016) with high affinity (Clausen et al., 2012). Importantly, VAR2CSA does not adhere to other organs in the body (Salanti et al., 2004). Subsequent studies revealed that various types of tumor cells also express CS similar to placental cells, thereby enabling VAR2CSA to specifically recognize and adhere to these tumor cells (Clausen et al., 2016; Agerbæk et al., 2018b; Wang et al., 2021). Salanti et al. demonstrated that VAR2CSA can specifically recognize and adhere to a variety of tumor cells *in vivo*, such as prostate cancer, breast cancer, and melanoma, significantly inhibiting tumor progression (Salanti et al., 2015). Moreover, VAR2CSA was shown to effectively bind to the anti-tumor drug Hemiasterlin, enabling targeted delivery to tumor cells and exerting an anti-tumor effect (Salanti et al., 2015). Due to its high specificity for tumor cells, VAR2CSA holds promise in the enrichment of circulating tumor cells in liquid biopsy of pancreatic, liver, and prostate tumors (Agerbæk et al., 2018a). Zhou et al. produced an imitation platelet nanoparticle containing VAR2CSA, exploiting the tumor-homing and metastasis-targeting properties of activated platelets and the tumor-specific binding ability of VAR2CSA. This approach successfully delivered chemotherapy drugs to tumor cells and effectively inhibited the growth of both *in situ* and metastatic tumors (Zhou et al., 2021). In addition, exosomes isolated from *Plasmodium*-infected mice were found to promote the expression of miRNA 16/322/497/17 in epithelial cells, reducing VEGFR2 expression, thus impacting tumor angiogenesis and inhibiting tumor progression in LLC mouse model (Yang et al., 2017). Further research on the anti-tumor components of *Plasmodium* will play a vital role in cancer screening and targeted therapy.

The anti-tumor research of *Plasmodium* has developed a balance between the attenuation of parasite strains and the targeting of tumor cells. However, most of the current studies only revealed the anti-tumor phenomenon, and the detailed mechanism is still unknown. In addition, there is a lack of research on the anti-tumor components of *Plasmodium*, and almost no other anti-tumor components have been focused on except VAR2CSA. More importantly, it is still not clear whether the *Plasmodium* can exert as good anti-tumor effect in humans as the animal test results. But fortunately, China has approved and initiated three single-arm clinical trials in the field of *Plasmodium* immunotherapy for advanced breast and liver cancers (NCT03474822), advanced cancers (NCT03375983), and advanced lung cancer (NCT02786589). Although the results of these trials have not yet been released, they are eagerly anticipated (Chen et al., 2021).

## 4 T. cruzi

**Figure 3:**
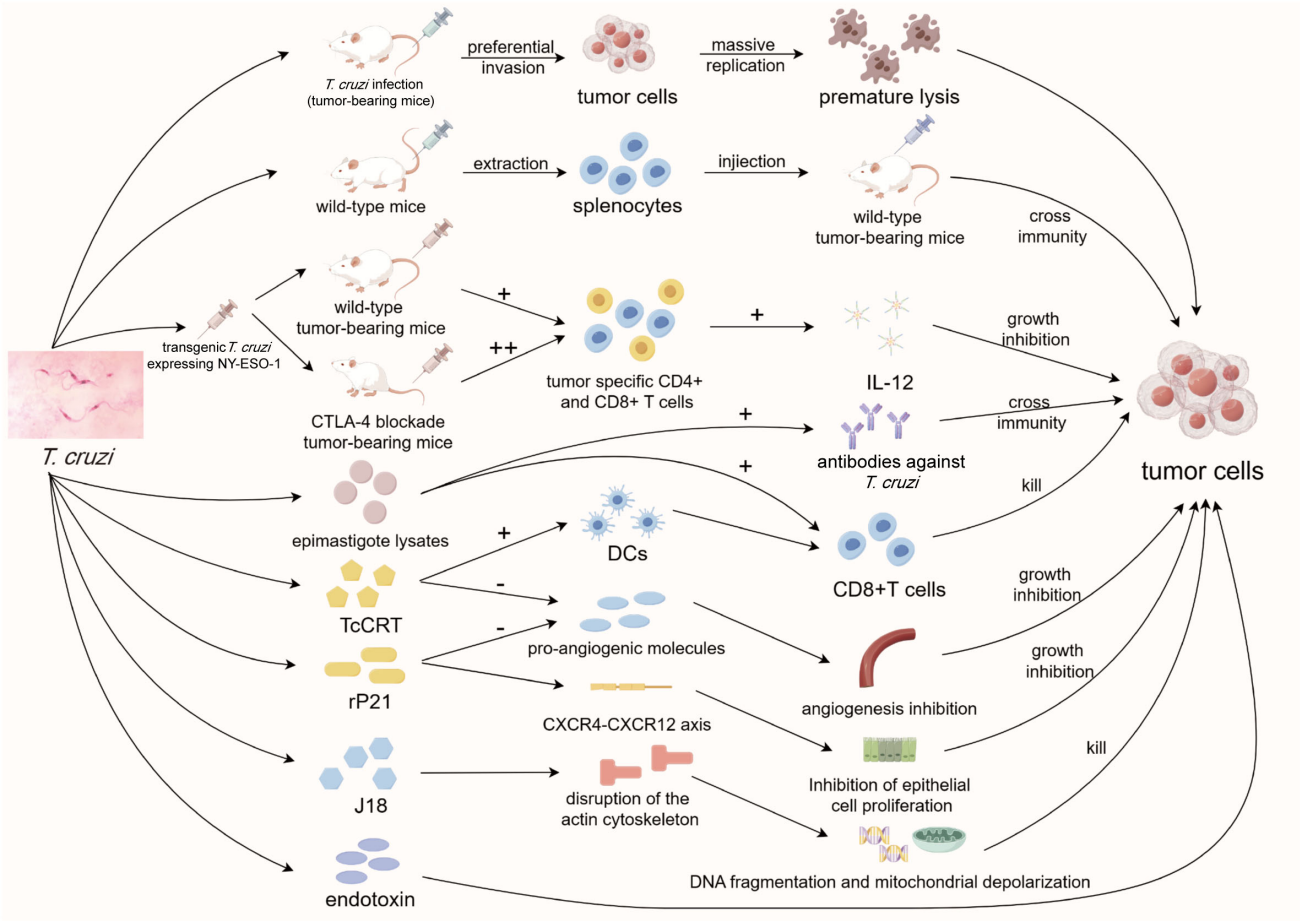
Diagram of anti-tumor mechanisms of *T. cruzi*. *T. cruzi* strains can kill tumor cells either by preferentially invading tumor cells or by activating T cells. More importantly, multiple specific components of TcCRT exert anti-tumor effects in multiple aspects. As a representative, TcCRT not only promotes antigen presentation of dendritic cells, but also plays an important role in anti-angiogenesis. *T. cruzi, Trypanosoma cruzi*; CTLA-4, cytotoxic T-lymphocyte antigen 4; CD4/8, cluster of differentiation 4/8; IL-12, interleukin-12; DCs, dendritic cells; TcCRT, *T. cruzi* calreticulin; rP21, recombinant P21; CXCR, C-X-C motif chemokine receptor.

Chagas disease, caused by the pathogen *T. cruzi*, primarily affects 21 countries in Latin America and is characterized by cardiomyopathy and arrhythmia as common symptoms (Pérez-Molina and Molina, 2018). The exploration of potential anti-tumor properties of *T. cruzi* dates back a century and has made significant progress. In the mid-20th century, two Soviet scientists discovered and reported the potential anti-tumor properties of *T. cruzi* extracts (Klyueva and Roskin, 1946), providing a novel approach to tumor treatment. *T. cruzi* and tumors both exhibit rapid division and immune evasion mechanisms within the human body, and the preferential invasion of tumor cells by *T. cruzi* has been extensively documented, leading to a certain degree of competition between them (Kallinikova et al., 2001; Ouaissi and Ouaissi, 2005). Furthermore, similar to protozoa mentioned above, some antineoplastic drugs and anti-*T. cruzi* medications have demonstrated therapeutic effects against both tumors and *T. cruzi* (Chennamaneni et al., 2009). Given this evidence, the anti-tumor potential of *T. cruzi* is worthy of further exploration (**Figure 3**).

### 4.1 Anti-tumor effects of *T. cruzi* infection

Building upon earlier findings, subsequent studies have aimed to unravel the anti-tumor mechanisms of *T. cruzi* (Jedeloo et al., 1950). Hatcher et al. demonstrated that cytotoxic responses against P-815 and YAC tumor cells were efficiently activated in mice infected with *T. cruzi* (Hatcher and Kuhn, 1981). Turning to the 21st century, more detailed experiments have since been conducted, taking advantage of improved experimental techniques and conditions. In the presence of DMH (1,2 dimethylhyadrazine), a drug that stimulates the development of colon adenocarcinoma, mice chronically infected subcutaneously with *T. cruzi* had a two-fold lower incidence of colon adenocarcinoma compared to a control group (Oliveira et al., 2001). This preliminary result confirmed the anti-tumorigenesis effect of *T. cruzi*. Additionally, *T. cruzi* displayed a preference for invading the MDA-MB-231 breast cancer cell line, impacting the proliferation and migration of tumor cells, ultimately leading to the early lysis and death of these cells in a triple-negative breast cancer cell invasion assay (Azevedo Silveira et al., 2023).

To enhance the precision of the immune response stimulated by *Trypanosoma* to target tumor cells, researchers have endeavored to genetically modify *T. cruzi*, enabling it to express the cancer testis antigen NY-ESO-1 while greatly reducing its toxicity (Junqueira et al., 2011). In mice intraperitoneally infected with the transgenic parasites, NY-ESO-1-specific CD4^+^ and CD8^+^ T cells were generated, triggering a range of MyD88- and IL-12-dependent tumor-specific humoral and cellular immune responses. This successfully inhibited tumor progression and prolonged the survival time of the infected mice (Junqueira et al., 2011). Furthermore, the blockade of CTLA-4 in mice intraperitoneally infected with transgenic *T. cruzi* enhanced the anti-tumor immune process by promiting the activation of NY-ESO-1 specific CD8^+^ T cells, increasing their IFN-γ expression, and enhancing their infiltration into the TME (Dos Santos et al., 2015). This suggests that transgenic *T. cruzi* can be used in combination with clinically employed immune checkpoint inhibitors to improve anti-tumor efficacy. It is evident that transgenic attenuated *T. cruzi* holds promising potential in the field of tumor immunotherapy, yet further research is needed to establish the technical basis for its clinical translation.

### 4.2 Anti-tumor ability of *T. cruzi* components

As early as 1948, researchers injected *T. cruzi* endotoxin directly into tumor-bearing mice, successfully inhibiting the growth of various types of tumors (Hauschka and Goodwin, 1948). Studies involving the lysates of different genetic groups of *T. cruzi* strains have revealed their notable anti-tumor capabilities (Sheklakova et al., 2003; Batmonkh et al., 2006). However, the significant side effects associated with these treatments hinder their direct use in cancer therapy. Therefore, there is a need to either purify these components or identify milder *T. cruzi* elements as alternatives. Subsequently, researchers extracted splenocytes from mice infected with *T. cruzi* lysates and injected them into tumor-bearing mice, resulting in a substantial inhibition of tumor progression (Zenina et al., 2008). This suggests that *T. cruzi* components do not necessarily need to be used directly within the body, but can employ activated immune cells to indirectly combat tumors, introducing a new application concept. Interestingly, splenocytes specifically targeting *T. cruzi* can simultaneously act on tumor cells, indicating that *T. cruzi* and tumor cells may share some antigens, leading to cross-immunity between them. Recently, researchers have successfully stimulated strong CD4^+^ and CD8^+^ T cell-mediated humoral and cellular immunity against tumors by injecting *T. cruzi* epimastigote lysates intraperitoneally. It has also been demonstrated that anti-*Trypanosoma* antibodies successfully cross-interact with breast and colon tumor cells (Ubillos et al., 2016). Collectively, these findings lay the theoretical foundation for subsequent studies on the anti-tumor effects of *T. cruzi* components.

Calreticulin (CRT) is a widely occurring protein in various organisms, including humans and protozoa, serving mainly as a chaperone and calcium buffer to ensure the correct folding of proteins in the endoplasmic reticulum (Fucikova et al., 2021). Apart from its role in the endoplasmic reticulum, calreticulin participates in multiple processes within the nucleus, cell membrane, and even extracellular regions (Michalak et al., 2009). It has been confirmed that calreticulin assists *T. cruzi* in invading host cells, contributing to immune evasion. Moreover, it exhibits anti-tumor properties (Ramírez et al., 2011). *T. cruzi* calreticulin (TcCRT) translocates from the endoplasmic reticulum to the parasite surface (RamÃ Rez-Toloza et al., 2015) and binds to component C1 to inhibit the classical complement pathway. It mimics an apoptotic cell marker to induce host cells to endocytose the parasite (Rimoldi et al., 1989; Álvarez et al., 2020), thereby realizing its dual purpose of evading host immunity and promoting invasion into cells (Ramirez et al., 2012). Notably, TcCRT or its N-terminal has been shown to inhibit capillary growth in rat aortic ring assays and hinder capillary morphogenesis in human umbilical vein endothelial cells *in vitro* (López et al., 2010). The N-terminal of *T. cruzi* calreticulin has the potential to directly interact with epithelial cells, inhibiting pro-angiogenic molecules, thereby resisting tumor angiogenesis, reducing oxygen and nutrient supply to tumors, and increasing the accumulation of metabolic waste products. This, in turn, induces cell apoptosis and inhibits tumor progression (Ramírez-Toloza et al., 2016). Additionally, dendritic cells invaded by *T. cruzi* can present TcCRT-derived antigen proteins to cytotoxic T lymphocytes, which may cross-react with tumor-specific antigens to also achieve the goal of tumor clearance (Ramírez-Toloza et al., 2016). The anti-angiogenesis of TcCRT has been confirmed in numerous animal experiments (Aguilar-Guzmán et al., 2014; Abello-Cáceres et al., 2016; Sosoniuk-Roche et al., 2020), highlighting its low toxicity and strong anti-tumor potential. These functions illustrate the interaction of *T. cruzi* with host immune defense strategies and potentially represent a coevolutionary adaptation for long-term interactions with the host.

In addition to calreticulin, *T. cruzi* possesses other specific components capable of exerting anti-tumor effects. In 2017, researchers found that the recombinant P21 (rP21) protein derived from *T. cruzi* directly acts on epithelial cells and interacts with CXCR4 to influence the CXCR4-CXCR12 axis *in vitro*. This interaction, along with its effect on the expression of angiogenesis-related genes, such as MMP9, sFlt-1, and VEGFR1, inhibits epithelial cell proliferation, and reduces angiogenesis (Teixeira et al., 2017). Subsequent experiments with rP21 on the triple-negative breast cancer cell line MDA-MB-231 *in vitro* demonstrated that rP21 itself is non-toxic and can inhibit the proliferation, migration, and invasion of tumor cells by adhering to them and reducing the expression of MMP-9 and CXCR4 (Borges et al., 2020). Another component, J18, a recombinant protein based on the surface molecule gp82 of *T. cruzi*, was confirmed to promote the disruption of the actin cytoskeleton in the tumor cell line Tm5 and affect a series of apoptosis-associated processes including DNA fragmentation, mitochondrial depolarization, and caspase-3 activity, thus inducing apoptosis in tumor cells (Atayde et al., 2008). Some components of *T. cruzi* exhibit good immunogenicity with minimal toxicity, making them suitable as immune adjuvants in tumor vaccines. Junqueira et al. found that glycoinositolphospholipids and CpGs oligodeoxynucleotides derived from *T. cruzi* could assist anti-tumor vaccine. By activating Toll-Like Receptor (TLR)4 and TLR9, they promote specific anti-tumor immune responses, including CD4^+^ and CD8^+^ T cell responses and IFN-γ responses in a melanoma mouse model (Junqueira et al., 2012). In summary, *T. cruzi* components primarily achieve their anti-tumor effects through non-immune pathways, and further research is needed to isolate, purify, and apply these effective components.

As the earliest protozoa to conduct anti-tumor research, *Trypanosoma* has made slow progress in anti-tumor research in recent years due to its lower prevalence than *T. gondii* or *Plasmodium*. *Trypanosoma* has a strong pathogenicity, and the persistent inflammation caused by its long-term infection can lead to accelerated cell death and regeneration, increased DNA damage and oncogene expression probability, thereby promoting the occurrence of cancer. Its correlation with gastrointestinal, gynecological, and hematological system tumors has been partially confirmed (Ribeiro Franco et al., 2023). Therefore, the toxic attenuation treatment of *Trypanosoma* is essential for its application in anti-tumor therapy, but unfortunately, there is still insufficient research on toxicity attenuation of *T. cruzi*, and the side effects or harm of its strains and components on the host cannot be effectively controlled. In addition, like other protozoa, due to limited conditions, current research is limited to animal experiments such as mice, and its anti-tumor efficacy cannot be observed in humans. Therefore, the effectiveness of many research results still needs further verification.

## 5 Other types of protozoa

In addition to the aforementioned protozoa, research into the anti-tumor potential of other protozoa remains relatively limited. *Leishmania*, the causative agent of leishmaniasis, is transmitted by female sandflies and is mainly endemic in seven countries, including Brazil, Ethiopia, and India. It can cause a wide range of symptoms, from mild skin lesions to severe internal organ damage (Burza et al., 2018), depending on the species. Sphingomyelin, a lipid found in cell membranes and intracellular regions, plays a crucial role in regulating cellular processes such as proliferation, differentiation, and apoptosis (Maceyka and Spiegel, 2014). After treating Sarcoma 180 cells with *Leishmania* sphingomyelin *in vitro*, reactive oxygen species production and caspase activation were regulated, resulting in up-regulation of pro-apoptotic molecules (Bad, Bax and P53) and down-regulation of anti-apoptotic molecules (Bcl-2 and PARP) (Das et al., 2015b). This treatment also inhibited the production of pro-angiogenic factors such as VEGF, CD34, and Ang-2, affecting the cell division cycle and thus impacting tumor growth (Das et al., 2015a). In addition, injection of attenuated Leishmania strains into tumor-bearing mice not only induced a strong Th1 immune response and the secretion of IFN-γ to combat tumors but also had minimal pathogenic effects on the mice (Caner et al., 2020), revealing the potential of attenuated *Leishmania* strains in anti-tumor applications.

*Eimeria* is a common apicomplexan parasite which can infect a variety of vertebrates, especially poultry, causing severe disease and economic losses (Adl et al., 2005). One of its constituent proteins, stiedae oocyst soluble protein, can induce a series of robust immune responses in tumor-bearing mice by intraperitoneal injection. This includes increased expression of CD80, CD86, MHC I, and MHC II on dendritic cells, elevated levels of CD107 and IFN-γ in CD8^+^ T cells and NK cells, as well as inhibited expression of molecules related to metastasis and angiogenesis, ultimately inhibiting tumor progression and prolonging survival time (Huang et al., 2021).

## 6 Discussion

Cancer, especially in advanced stages, remains a formidable global health challenge with uncontrolled prevalence and high mortality. Protozoa, traditionally seen as human and animal parasites, have shown potential in inhibiting cancer progression in numerous studies. This article reviews the research results on various protozoan parasites (*T. gondii*, *Plasmodium*, *T. cruzi*, etc.) and their role in anti-tumor therapies over the past decades. Upon invading the host, these protozoa can activate the immune system, reversing the immunosuppressive state of the TME and orchestrating various immune cells and molecules to inhibit tumor onset, progression, and recurrence. Moreover, they can induce tumor cell apoptosis through a variety of non-immune mechanisms, such as anti-angiogenesis and promotion of DNA fragmentation and mitochondrial depolarization. While *Plasmodium* research primarily focuses on the anti-tumor effects of the parasite strain, *T. cruzi* research centers mainly on its various components, and *T. gondii* has made much progress in both aspects. However, these investigations on protozoan anti-tumor properties have mostly been limited to *in vitro* or animal experiments with a surface-level understanding of the mechanisms involved. Systematic human trials have yet to be conducted due to experimental and ethical constraints in the past. Therefore, potential of protozoa in cancer therapy and their mechanisms in the human body remain inconclusive, necessitating further extensive experimentation.

Given the varying life cycles and anti-tumor mechanisms of different protozoa, and the potential impact on different types of tumors and humans, the factors affecting the anti-tumor efficacy of protozoa in humans are complex. To apply protozoan anti-tumor therapy in clinical practice, the compatibility between different protozoa and various tumors must be explored in real-world human settings. Hence, clinical trials within the scope of safety and ethics are necessary. At present, there are well-established cancer therapies in clinical practice, including traditional approaches such as radiotherapy, chemotherapy, and surgery, as well as emerging targeted therapy and immunotherapy. Protozoan treatment, as a novel approach, can be combined with mature therapies such as chemotherapy or immune checkpoint inhibitors. While confirming the anti-tumor efficacy of protozoan treatment, their combination with existing therapies may lead to improved treatment outcomes. It is worth mentioning that as early as the end of last century, some studies confirmed that *T. gondii* extract can reverse the multidrug resistance of gastric cancer cells (Varga et al., 1999), confirming the feasibility of this approach.

Protozoa, as parasitic organisms, inherently pose a threat to human health. Patients with cancer often have compromised or incomplete immune systems due to the influence of tumor cells, making them more susceptible to the negative effects of parasites, potentially exacerbating cachexia (Hatter et al., 2018). Although the anti-tumor effects of protozoa have been confirmed, their side effects cannot be ignored and must be carefully considered. Some researchers have attempted to reduce the toxicity of protozoa, either through genetic or radiative means, while preserving their invasion capabilities. However, these attempts have been confined to animal experiments, and their effects on patients with cancer are unknown. In addition, attenuated protozoa may only stimulate the immune system for a limited period, potentially necessitating continuous, long-term protozoa treatment to achieve the desired anti-tumor effects, thereby imposing a greater burden on patients. Interestingly, some drugs with both anti-tumor and anti-parasitic effects have also been identified, and using these drugs to mitigate the side effects of protozoa and assist anti-tumor therapy may be a viable approach.

Beyond the lack of clinical trials, numerous aspects of protozoan anti-tumor therapy require further exploration. While the phenomenon of inhibiting the occurrence, progression, and recurrence of various tumors by different protozoa has been substantiated through multiple means, the underlying mechanisms, particularly non-immune mechanisms, remain incompletely elucidated. A comprehensive understanding of these mechanisms is crucial for the successful application of protozoan anti-tumor therapy in clinical cancer treatment. Moreover, aside from VAR2CSA in *Plasmodium* and CRT in *T. cruzi*, there are likely many other genes, proteins, or other components with anti-tumor potential awaiting discovery in each type of protozoa. Advances in experimental conditions and novel technologies, including multi-omics and bioinformatics analysis techniques, can aid in screening and verifying the anti-tumor functions of these components.

Overall, research on the anti-tumor effects of protozoa has made significant progress in the past few decades, underscoring their promising potential. However, to successfully apply protozoa in clinical cancer therapy, substantial further research is necessary to optimize treatment efficacy, minimize side effects, and validate their effectiveness in cancer patients. It is certain that protozoa hold promise for making a significant contribution to cancer therapy in the future.

## Author contributions

ZZ: Visualization, Writing – original draft, Writing – review & editing, Resources. XL: Writing – original draft, Writing – review & editing, Resources. DZ: Writing – review & editing, Resources, Writing – original draft. XD: Writing – review & editing, Visualization. QL: Writing – review & editing. X-bL: Writing – review & editing. JZ: Writing – review & editing. YL: Writing – review & editing, Visualization. HZ: Conceptualization, Supervision, Writing – review & editing, Funding acquisition. JD: Funding acquisition, Writing – review & editing, Conceptualization, Supervision.
